# SARS-CoV-2 transmission in teenagers and young adults in Fútbol Club Barcelona’s Multidisciplinary Sports Training Academy

**DOI:** 10.1007/s00431-023-04880-x

**Published:** 2023-03-14

**Authors:** María Hernández-García, Quique Bassat, Victoria Fumado, Gil Rodas, Ramon Pi, Maite Miranda-Garcia, Mònica Girona-Alarcón, Martí Català, Sergio Alonso, Enrique Alvarez-Lacalle, Daniel López, Maria Melé-Casas, Gemma Pons-Tomas, Mariona F. de Sevilla, Elisenda Bonet-Carne, Claudia Fortuny, Aleix García-Miquel, Cristina Jou, Cristina Adroher, Joana Claverol, Marta Cubells, Anna Codina, Daniel Cuadras, Eduard Gratacós, Pedro Brotons, Carmen Muñoz-Almagro, Clara Prats, Juan José García-García, Iolanda Jordan

**Affiliations:** 1 Paediatrics Department, Hospital Sant Joan de Déu Barcelona, Passeig Sant Joan de Déu, Number 2, Barcelona, Esplugues de Llobregat 08950 Spain; 2 ISGlobal, Barcelona Institute for Global Health, Barcelona, Spain; 3 Centro de Investigación Biomédica en Red de Epidemiología Y Salud Pública (CIBER-ESP), Instituto de Salud Carlos III, Madrid, Spain; 4 Centro de Investigação Em Saúde de Manhiça (CISM), Maputo, Mozambique; 5 Catalan Institution for Research and Advanced Studies, ICREA, Barcelona, Spain; 6 Infectious Diseases Department, Hospital Sant Joan de Déu Barcelona, Barcelona, Spain; 7 Infectious Diseases and Microbiome, Institut de Recerca Sant Joan de Déu (IRSJD), Barcelona, Spain; 8 Faculty of Medicine and Health Sciences, Universitat de Barcelona, Barcelona, Spain; 9 Sports Medicine Unit, Hospital Clinic and Hospital Sant Joan de Déu Barcelona, Barcelona, Spain; 10 Medical Department, Futbol Club Barcelona, Barça Innovation Hub, Barcelona, Spain; 11 Fundació Sant Joan de Déu, Barcelona, Spain; 12 Paediatric Intensive Care Unit, Hospital Sant Joan de Déu Barcelona, Barcelona, Spain; 13 Nuffield Department of Orthopaedics, Rheumatology and Musculoskeletal Sciences (NDORMS), University of Oxford, Oxford, UK; 14 Computational Biology and Complex Systems (BIOCOM-SC), Department of Physics, Universitat Politècnica de Catalunya, Castelldefels, Spain; 15 BCNatal Fetal Medicine Research Center, Hospital Clinic and Hospital Sant Joan de Déu Barcelona, Barcelona, Spain; 16 Institut d Investigacions Biomèdiques August Pi I Sunyer (IDIBAPS), Barcelona, Spain; 17 Universitat Politècnica de Catalunya • BarcelonaTech, Barcelona, Spain; 18 Department of Pathology and Biobank, Hospital Sant Joan de Déu Barcelona, Barcelona, Spain; 19 Centro de Investigación Biomédica en Red de Enfermedades Raras (CIBER-ER), Instituto de Salud Carlos III, Madrid, Spain; 20 Strategic Planning and Management Control, Hospital Sant Joan de Déu Barcelona, Barcelona, Spain; 21 Statistics Department, Fundació Sant Joan de Déu, Barcelona, Spain; 22 Department of Medicine, Universitat Internacional de Catalunya, Barcelona, Spain; 23 RDI Microbiology Department, Hospital Sant Joan de Déu Barcelona, Barcelona, Spain

**Keywords:** COVID-19, SARS-CoV-2, Adolescent, Transmission, Sports, Youth sports

## Abstract

Most studies, aimed at determining the incidence and transmission of SARS-CoV-2 in children and teenagers, have been developed in school settings. Our study conducted surveillance and inferred attack rates focusing on the practice of sports. Prospective and observational study of those attending the sports facilities of Fútbol Club Barcelona (FCB), in Barcelona, Spain, throughout the 2020–2021 season. Participants were young players (from five different sports) and adult workers, who belonged to stable teams (shared routines and were involved in same quarantine rules). Biweekly health questionnaires and SARS-CoV-2 screening were conducted. From the 234 participants included, 70 (30%) both lived and trained in the FCB facilities (Recruitment Pathway 1;RP1) and 164 (70%) lived at their own household and just came to the facilities to train (RP2). During the study, 38 positive cases were identified; none had severe symptoms or needed hospitalization. The overall weekly incidence in the cohorts did not differ compared to the one expected in the community, except for 2 weeks when an outbreak occurred. The attack rate (AR) was three times higher for the participants from RP1, in comparison to those from RP2 (*p* < 0.01). A Basketball team showed a significant higher AR.

*  Conclusion*: Physical activities in stable teams are not related to an increased risk of transmission of SARS-CoV-2, since there were the same observed cases than expected in the community. The risk is higher in indoor sports (Basketball vs. Football), and in closed cohort living settings (RP1 vs. RP2). The fulfilment of preventive measures is essential.

**Table:** 

**What is Known:** *• Despite the low numerical impact caused in paediatric hospitalizations during COVID-19 pandemic, the social impact has been maximum.* *• The transmission potential in children and teenagers is limited, and it had been widely demonstrated in school settings.*	
**What is New:** *• Group physical activities in children and teenagers are not also related to an increased risk of transmission of SARS-CoV-2, when preventive measures, such as washing hands, and screening protocols are applied.* *• Routine and semi-professional sports activities seem safe environments to promote during this pandemic.*

**Supplementary Information:**

The online version contains supplementary material available at 10.1007/s00431-023-04880-x.

## Introduction

The literature generally describes a lower incidence, transmission and severity of SARS-CoV-2 in the paediatric population[1–3]. The emergence and spread of new variants, such as B.1.1.529 (Omicron), threaten the findings observed during the first 2 years of the pandemic[4], and the real repercussion on children and teenagers remains to be seen.

Despite the low impact caused in paediatric hospitalizations, the social impact has been maximum [5]. Based on data from other viral infections, it was hypothesized that children should be one of the main targets of confinement at the start of the pandemic [6–8]. Current data indicates the contrary, and there is ample evidence, for example, in the context of reopening of schools, that children’s transmission potential is limited [9, 10], also appearing to increase with age [11].

Most studies have been conducted in schools and in ages below 12–15 years [12, 13], with little investigation in sports [14, 15], an area in which it should be done more emphasis given its important contribution for overall health, mental health and school performance. During successive waves, most countries chose to limit sports activities, without good-quality data supporting such decisions, leading to deleterious effects on the youngers, limiting their learning and their ability to maintain a level of recommended physical activity [16].

One proposed solution for transmission containment is proactive screening before participation in sports. This is particularly common at the competition level [17]. Before vaccines were available, one approach involved IgG antibodies testing, with a positive serology implying protection, a method abandoned with widespread vaccination. Furthermore, the evolution of the infection, the longevity of the immune response [18] and the correlation of antibody levels with clinical protection remain insufficiently characterized to date [19]. The main useful diagnostic method has been nasopharyngeal PCR, followed by nasopharyngeal antigen and saliva PCR. The problem is that the sensitivity and specificity of the tests available on a large scale remain variable[20], which makes it difficult to establish standardized screening protocols in the sports field.

Conducting a study within the facilities of Fútbol Club Barcelona (FCB), where many teenagers and young people live or meet routinely, provided an unprecedented opportunity to determine transmission of SARS-CoV-2 in the world of semi-professional sports. FCB hosts outdoors and indoors sports, with different contact between players, which could lead to distinct transmission patterns. Furthermore, the fact that participants shared both sports activities and living spaces offered a good setting to study what can happen with the maintenance of sports activities in other areas.

The aim of the study was to carry out SARS-CoV-2 surveillance and infer attack rates among adolescents in the FCB facilities, trying to find factors related to transmission.

## Materials and methods

Prospective, observational and longitudinal study of those attending FCB sports facilities, in Barcelona, Spain, throughout the 2020–2021 season (August 1st 2020–June 16th 2021).

Included participants were teen players (12–17 years old), young adult players (18–23 years old) and adult workers. Participants who did not sign the informed consent or unwilling to ensure adequate follow-up were excluded.

### Study development

Two recruitment pathways (RP) were considered: RP1 included participants who lived, studied and trained in the facilities (a residential and sports area called “Masia”); RP2 included participants who lived in their own homes, and just went to train and study in the facilities. Seven complete teams were tracked during the whole course: Outdoor Football, Men: (1) Senior youth A, (2) Senior youth B, (3) Junior youth A; Outdoor Football, Women: (4) Reserve, (5) Senior youth; Basketball: (6) Reserve, (7) Senior youth. In each of them, there were participants who belonged to RP1 and RP2. In addition to these seven teams, others were studied, although not all their components wanted to participate.

A survey was conducted upon recruitment including clinical and epidemiological data. During follow-up, a biweekly health questionnaire was applied enquiring about symptoms and possible COVID-19 contacts. A nasopharyngeal (NP) PCR was performed every 15 days, together with a weekly saliva sample (stored as backup sample). Serologies were done upon enrollment and every 3 months.

From January to June 2021, nasopharyngeal antigens (NP Ag) replaced PCRs, a change applied in parallel to its wider implementation, facilitating a faster turnaround of results. Considering the possibility of false negatives, testing frequency was increased to a weekly basis. However, if participants reported symptoms, or were a contact of a positive case, an additional NP PCR was performed.

When a positive case was detected in a team (considered stable sport-specific groups, made up of the same participants throughout the course, following the same routines and isolation protocols), all its components were considered close contacts, so they were studied and immediately confined (see more information in the Supplementary file).

According to Spanish legislation, the use of face masks was compulsory, and it was recommended to maintain a minimum interpersonal distance and to wash hands regularly. However, players within the same team were allowed to remove masks during training or in their houses/rooms. Other preventive measures were applied, such as daily temperature control (see more information in the Supplementary file).

The first vaccination campaign against SARS-CoV-2 in Spain began on December 27th 2020. The campaign for the population between 16 and 29 years old started on June 30th 2021. During the study, 23 (36%) adult workers received at least one vaccine.

### Definitions

A positive case was defined as a participant with a positive NP PCR/Ag, in the absence of IgG. At enrollment, cases with positive PCR but also positive IgG were considered past infections and were not included as positive cases. The only evidence of a following seroconversion in the vaccinated participants was not criteria for the definition of a positive case.

Cases with an indeterminate PCR were closely followed up by questionnaires and weekly PCR, and were considered negative unless a subsequent PCR resulted positive.

We could not distinguish between primary and secondary positive cases, due to the participants belonged to “crossed” cohorts: those belonging to RP1 or RP2 vs. each particular team independently of where they live.

### Outcomes

The primary outcome was to determine the attack rate (AR) in the different cohorts: RP1, RP2, and the seven complete teams. For each cohort, we defined the AR as the percentage of participants who provided at least one positive test during the study. We chose this method instead of widely used ones such as the secondary attack rate (SAR) due to the impossibility of explicitly defining the primary and secondary cases, given the stated crossed-nature of the cohorts.

$$\mathrm{AR}=\frac{\mathrm{Number}\;\mathrm{of}\;\mathrm{positive}\;\mathrm{cases}\;\mathrm{in}\;\mathrm{side}\;\mathrm{the}\;\mathrm{cohort}}{\mathrm{Number}\;\mathrm{of}\;\mathrm{participants}\;\mathrm{of}\;\mathrm{the}\;\mathrm{cohort}}100$$

With the aim of identifying when transmission was significant and led to local outbreaks, we determined the expected number of cases that would be found according to the surrounding incidence (see more information in the Supplementary file).

### Laboratory measurements

SARS-CoV-2 PCR in NP and saliva was performed according previous published[21]. A positive result was considered if at least two SARS-CoV-2 genes were detected, considering “indeterminate” when only one gene was detected.

Rapid Antigen Test used was from Roche®. IgG, IgA and IgM antibodies were determined using a Luminex system against the receptor-binding domain of the spike glycoprotein of SARS-CoV-2.

### Ethics

The study was approved by the Institutional Review Board and the Sant Joan de Déu Ethics Committee (PIC-200–20) and followed Helsinki Declaration recommendations. All participants or their legal guardians provided a written informed consent.

### Statistics

A Redcap-designed database was used and participant’s data were pseudo-anonymized. All athletes, except the professional teams, were offered participation, so no formal sample size calculation was conducted.

Chi-squared test and Fisher’s exact test were used for comparisons of categorical data; Student’s *t*-test and Mann–Whitney’s *U*-test for quantitative variables. To compare epidemiological and microbiological results at different times, the Wilcoxon signed-rank test was used. Odds ratio, determined with Fisher’s exact test and a 2 × 2 table (Matlab’s function), was used to assess the AR of a specific cohort in relation to all the others.

## Results

Of 283 eligible athletes and workers, 246 were initially recruited, of whom 234 were finally included (83% of participation). Eight participants were lost in the follow-up. From the 234 individuals, 170 were players (125 teen and 45 young adult; overall mean age of 16.4 years, SD = 2.14) and 64 adult workers (mean age 39.4 years, range 27–61, SD = 8.53). Seventy participants (30%) came from RP1 (lived in the Masia) and 164 (70%) from RP2 (lived in their own household). Participants played in five different sports: football (outdoor and indoor), basketball, handball and hockey. Overall, 141 (82.5%) players were males. Football had both men’s and women’s team; the rest of sports had only men’s team. Table 1 details the participants’ characteristics; Table 2 details sports and teams.

**Table 1:** Epidemiological general characteristics of the participants, positive cases and non-infected participants comparison

**Variable**	**All participants** **(*n*** **= 234)**	**Positive cases** **(*n*** **= 38)**	**Non-infected participants** **(*n*** **= 196)**	***p***
Gender, male	185 (79%)	34 (89%)	151 (77%)	0.125
Age, years	20.2 (12–61)	18.4 (12–50)	20·6 (12–61)	0.174
Ethnicity				**0.008**
Caucasian Indian-Asian Maghrebi-Arabians Latin-Americans Sub-Saharans Others	174 (74.4%) 2 (0.9%) 3 (1.3%) 13 (5.6%) 4 (1.7%) 38 (16.2%)	22 (57.9%) 1 (2.6%) 0 2 (5.3%) 3 (7.9%) 10 (26.3%)	152 (77.5%) 1 (0.51%) 3 (1.53%) 11 (5.6%) 1 (0.5%) 28 (14.3%)	
Previous disease	15 (6.4%)	2 (5.3%)	13 (6.6%)	0.75
Hospitalization during 2020				
	13 (5.6%)	2 (5.3%)	11 (5.6%)	0.93
Infections in previous 3 months				
Respiratory Gastroenteritis Other	2 (0.8%) 6 (2.6%) 5 (2.1%)	0 0 0	2 (1%) 6 (3%) 5 (2.5%)	0.53 0.27 0.31
Household inhabitants	3 (0–7)	4 (0–7)	3 (0–7)	0.21
House surface (m^2^)	131	171	123	0.37
Number of bathrooms in house				
	2 (1–6)	2 (1–4)	2 (1–6)	0.85
Sharing bedroom	84 (35.9%)	9 (23.7%)	75 (38.3%)	0.08
Adults > 65 years in household				
	1 (2.1%)	0	1 (2.4%)	0.72
Wearing masks in the street				
	215 (91.9%)	35 (92.1%)	180 (91.8%)	0.38
Transport used to reach campus				0.08
Walking/cycling Public transport Private transport Does not apply (lives there)	26 (11.1%) 33 (14.1%) 119 (50.8%) 56 (23.9%)	2 (5.3%) 4 (10.5%) 17 (44.7%) 15 (39.5%)	24 (12.2%) 29 (14.8%) 102 (52%) 41 (20.9%)	
Other suspected or confirmed COVID cases in household				
Adult Children	15 (6.4%) 7 (3%)	0 0	15 (7.7%) 7 (3.6%)	0.07 0.23

**Table 2:** Classification of the teams to which both players and adults belonged, including the description of the positive cases and the attack rate (AR) of each monitored team in the study

**Sport**	**Teams**			**Total participants**	**Positive cases** (% respect of all positive cases)	**Attack rate (AR)**
***7 complete monitored teams***						
Outdoor Football	Men	Senior youth A	Players	26	5 (13%)	14%
			Workers	9	0	
		Senior youth B	Players	24	8 (21%)	24%
			Workers	9	0	
		Junior youth A	Players	22	2 (5%)	8%
			Workers	4	0	
	Women	Reserve	Players	18	2 (5%)	10%
			Workers	3	0	
		Senior youth	Players	13	1 (3%)	8%
			Workers	0	0	
Basketball	Reserve		Players	12	1 (3%)	13%
			Workers	3	1 (3%)	
	Senior youth		Players	12	8 (21%)	67%
			Workers	3	2 (5%)	
***Total complete monitored teams***				***158***	***30 (79%)***	***19%***

***Rest of the participant but incomplete teams***						
Outdoor Football	Men	Junior youth B	Players	4	3 (8%)	NA
			Workers	0	0	
		Junior youth C	Players	6	0	NA
			Workers	0	0	
		Junior youth D	Players	3	0	NA
			Workers	0	0	
Indoor Football	Reserve			5	1 (3%)	NA
	Senior youth			1	0	NA
	Junior youth			1	0	NA
	Workers			0	0	NA
Basketball	Junior youth A		Players	4	1 (3%)	NA
			Workers	0	0	
	Junior youth B		Players	1	1 (3%)	NA
			Workers	0	0	
Handball	Reserve			3	0	NA
	Senior youth			2	1 (3%)	NA
	Junior youth A			3	0	NA
	Junior youth B			1	0	NA
	Workers			0	0	NA
Hockey	Reserve			4	0	NA
	Senior youth			4	0	NA
	Junior youth			1	0	NA
	Workers			0	0	NA
*Other workers*				33	1 (3%)	NA
***Total incomplete teams***				***76***	***8 (21%)***	***NA***

Note that the attack rate of the incomplete teams are not reported (*NA*, non-applicable), since the information of the whole team for the whole period was not available

Age ranges (in years old): Reserve team 18–23, Senior youth team 16–18, Junior youth team 14–16 (applicable to A and B teams) and 12–13 (applicable to C and D teams)

A total of 1986 NP PCR and 2777 NP Ag were obtained. Additionally, serological testing was conducted to 229, 118, 214 and 192 of participants, during the different scheduled time points. Eighty-two participants (35%) reported symptoms at least once in the health questionnaires.

### Positive cases

Altogether, 38 positive cases were identified: 34 players and 4 workers. Twenty-one came from the RP1 and 17 from the RP2. Fifteen out of 34 players (44%) had mild symptoms (fever and cough the most frequent), being the remaining 56% asymptomatic. However, most workers (75%) were symptomatic. None of the positive cases had severe symptoms or required hospitalization. All data on positive cases are described in Tables 1 and 2.

**Fig. 1:**
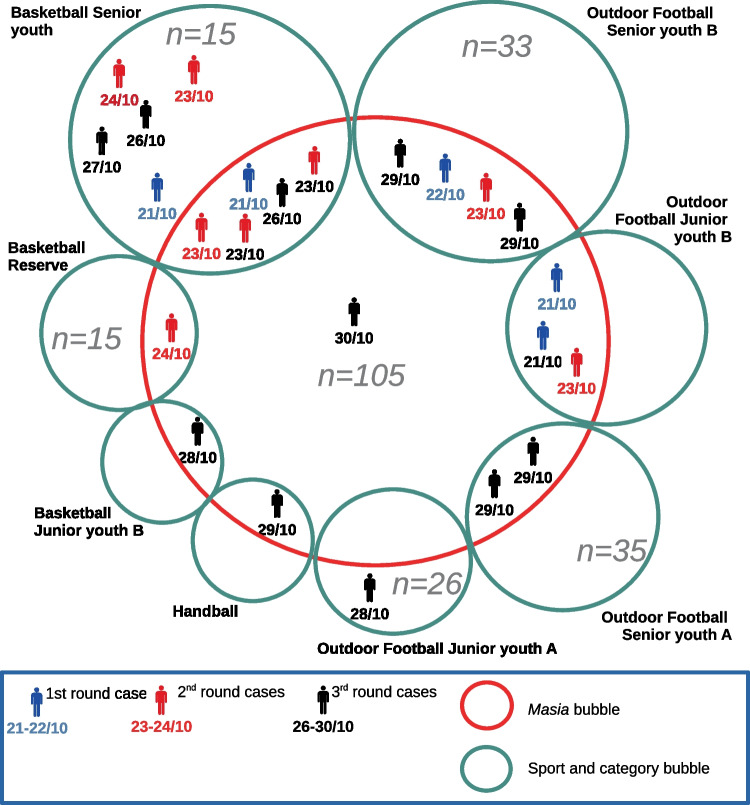
October 2020 positive cases outbreak (21st to 30th October). The cases are differentiated in three rounds according to the dates of the first positive test (PCR or Ag) or the first symptom, in order to show the temporal evolution (blue, cases dated between 21st and 22nd October; red, cases dated between 23rd and 24th October; black, cases dated between 26 and 30th October). They are included in their Sport and Category “Bubble” (teams, green circles) as well as in their household “bubble” (in or out the Masia, in the Masia being included in the red circle). The grey numbers indicate the size of the tracked cohorts. Absence of grey number indicates that such team was not included as a whole in the study (i.e. the participant was followed through the Masia)

In October 2020 (43rd–44th week of the year), there was an unexpected outbreak. Twenty-four positive cases were detected, most of them (75%) from RP1. Five initially positive cases were identified, as they presented symptoms or had positive tests first. Four of them lived in the Masia, although belonged to three different teams. Most of the positive cases belonged to basketball teams. All data is showed in Fig. 1.

The remaining 14 positive cases were either isolated or with transmission to a single person within the same team.

### Attack rates, expected incidence and infectivity

The mean of contacts studied from the same team for each positive case was 11 (IQR 14). The global AR of the participants from RP1 was 30%, being 10% of those from RP2. Among the seven monitored teams, the overall AR was 19%, being 35% the AR of the participants from RP1, and 13% the AR of those from RP2. The ARs of each monitored team are reported in Table 2.

The pairwise comparisons showed significant differences between the AR regarding the recruiting pathway, with an odds ratio (OR) of 3.7 [1.8–7.6], entailing that the AR was higher among participants from RP1 when compared with RP2. If we compare the AR of RP1 participants that lived in the Masia with regard to those that worked there, the difference was even higher (*p* < 0.01, OR 13.7 [1.8–107.1]). Furthermore, focusing on the seven monitored teams, we compared the AR among those that lived in the Masia with those that did not, obtaining a significant OR of 3.6 [1.6–8.2].

As for the monitored teams, the only one that showed a significant increase in its AR was the Basketball Senior youth, with an OR of 12.3 [3.8–39.7]. This significance was lost when the comparison was restricted to those from RP1, while it was maintained when comparing to those from RP2 (see more information in the Supplementary file).

**Fig. 2:**
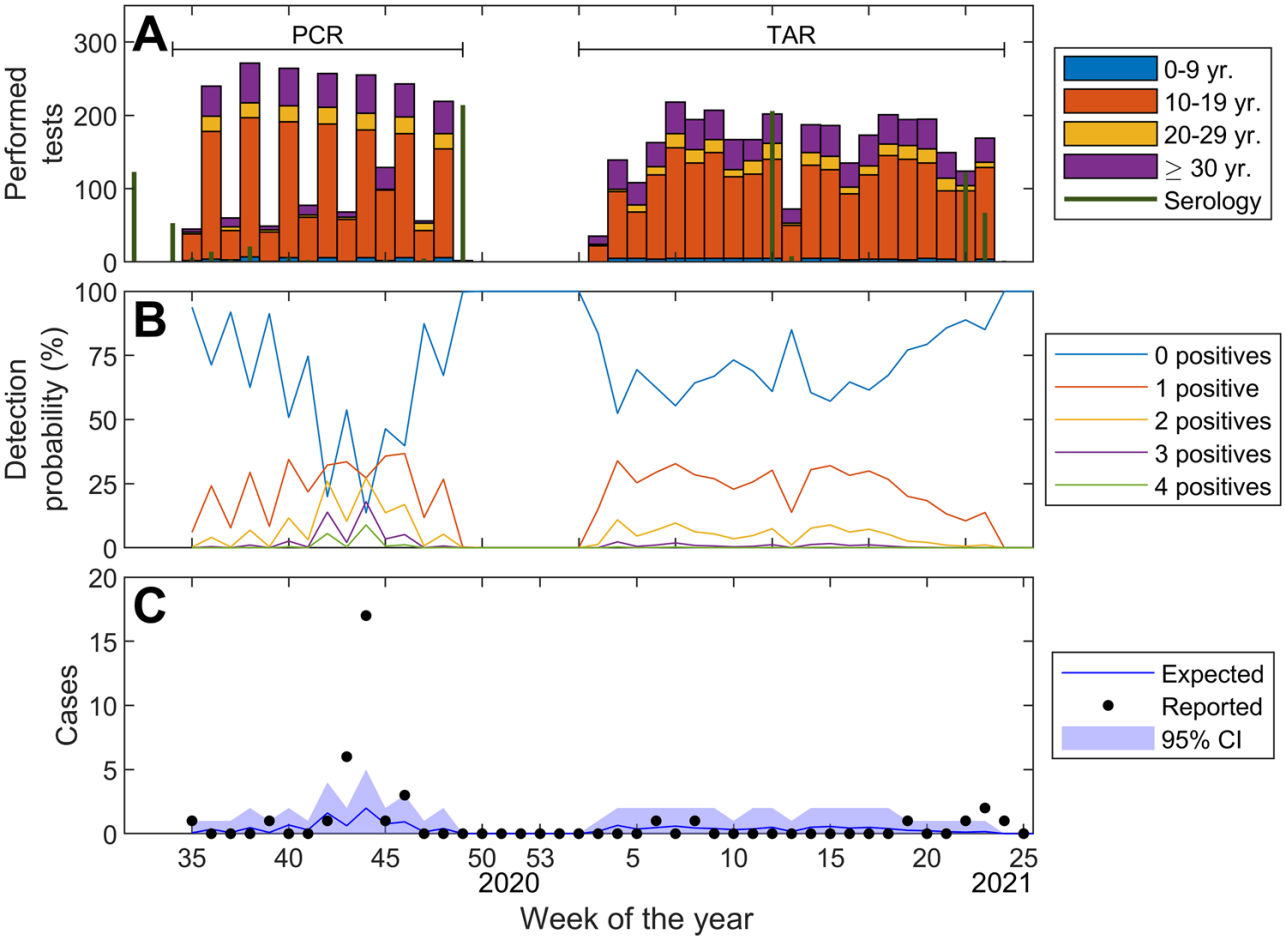
Expected and detected positive cases, and number of performed tests. **A** Number of PCR, Ag test and serologies performed during the study, per week and divided into age ranges. **B** Probability of detecting 0 to 4 positives each week, based on the tests performed. **C** Number of positive cases reported compared to those expected (taking as a reference the incidence of positive cases at the same moment in the surrounding areas of the FCB facilities), with 95% CI (for the expected cases)

The number of tests performed and the observed positive cases according to the detection probability in comparison to the surrounding areas are presented in Fig. 2. The figure shows that major outbreaks were rare, except for the one already mentioned. The total number of expected cases throughout the study was 16.2 [95%CI 10–23], being 38 the observed cases, therefore more than expected. However, recalculating without taking the outbreak into account, the expected cases were 13.6 [95%CI 8–20], and the observed cases were 14, which entails a non-significant difference.

Regarding infectivity, the PCRs performed remained positive for a median of 1.5 weeks (range 1–4). Having a persistently positive PCR more than 10 days was not associated with having symptoms, and neither to an increased infectivity to others.

### Seroconversion

Results from the baseline screening conducted to 225 individuals included no positive PCRs and 11 positive IgG, which were subsequently considered past infections. Only one of these 11 became re-infected, while maintaining the serologic positivity at the end of the study. From the other 10, only one lost the seroconversion.

Along the study, 35 from the 214 (16.3%) with initially negative baseline PCR and serology became infected; most of them (91.4%) presented seroconversion.

**Fig. 3:**
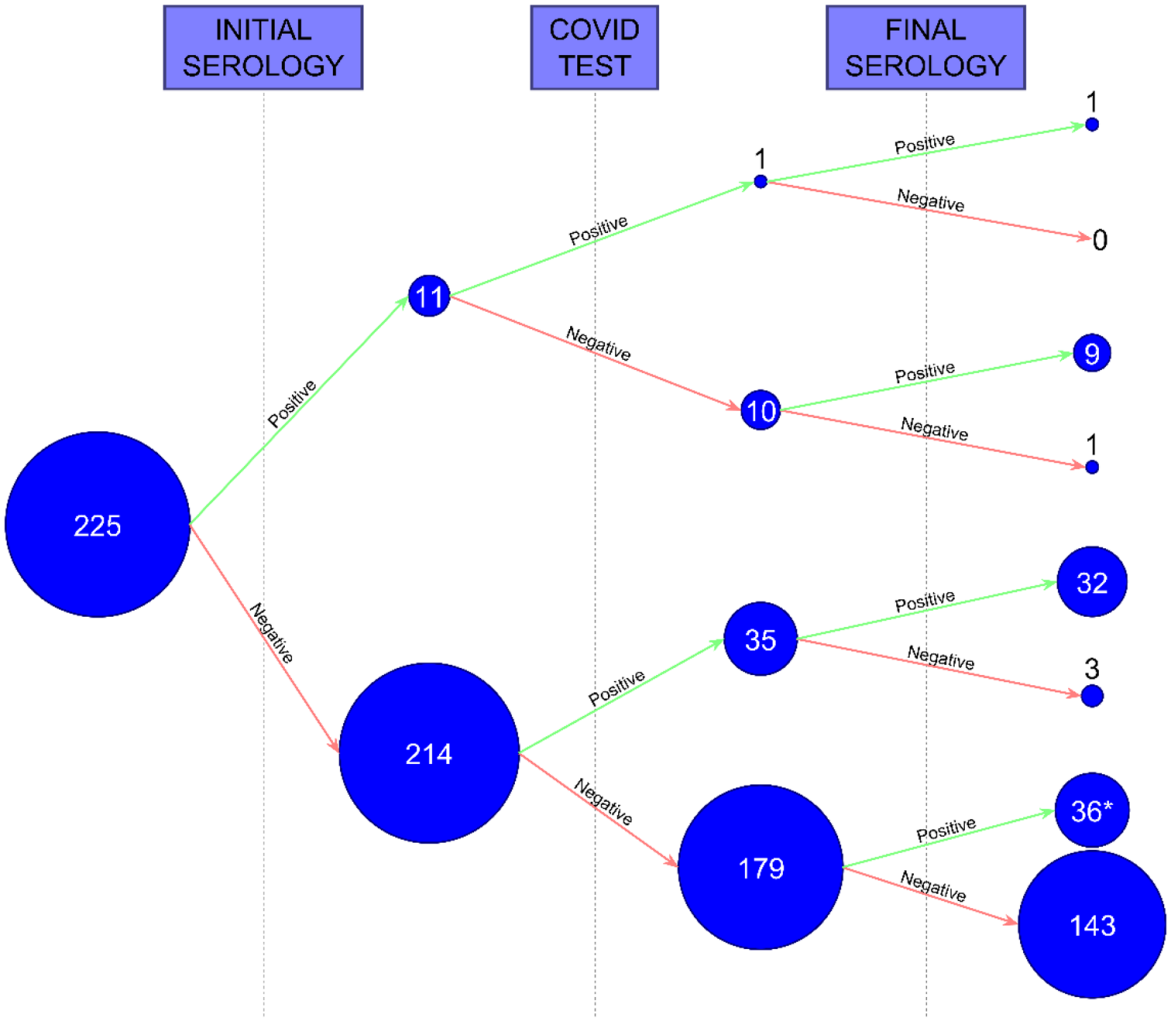
Description of the seroconversion throughout the study. Of the 234 participants, 225 were the ones who underwent both initial and final serology during the whole study. From the 179 participants, with an initial negative serology, for whom a positive SARS-CoV-2 result was never documented, 36 (20.1%) had seroconverted by the end of the study. *Of these 36 participants, 20 (55%) were vaccinated against SARS-CoV-2 during the study, so the positive IgG are explained by the immunization. From the other 16 participants: 8 (22.5%) of them did have a positive test, although it was done outside the study (through the primary care center or privately), and the remaining 8 (22.5%) had no data of a positive test either inside or outside the study

From the 179 participants for whom a positive SARS-CoV-2 result was never documented, 36 (20.1%) had seroconverted by the end of the study. Of them, 20 (55%) were vaccinated against SARS-CoV-2, so the positive IgG were explained by immunization. Of the other 16 participants: eight (22.5%) had a positive test, although it was done outside the study (e.g. primary care center); and the remaining eight (22.5%) had no data of a positive test, so those were the only ones considered infected although being asymptomatic and with negative tests. All these data are showed in Fig. 3.

## Discussion

During the first waves of the pandemic, several countries imposed strict restrictions on children, including closing of schools and limiting the practice of sports, since at that time they had been considered super-spreaders of SARS-CoV-2. However, there was not robust evidence supporting these restrictions, leading to discussion regarding the safe return to normal activities in schools and teams for the 2020–2021 season [22, 23].

Endorsing limitations in sports has had serious consequences on both mental and physical health [24]. Anxiety, depressive symptoms and increased suicidal ideation have been reported [5, 25]. Furthermore, a greater sedentary lifestyle has been described, which could lead to serious long-term outcomes, such as metabolic syndrome, obesity and diabetes [16].

One of the basis for these restrictions was that some evidence suggested an increased risk of transmission due to a higher excretion of microparticles during sports [26]. While this might be true in adults, it has not been demonstrated in younger people. In fact, our study shows that physical activities in stable teams are not related to an increased risk of transmission, since only one outbreak occurred, and the rest of the season there were the same observed cases than expected cases in the community. Actually, there was no statistically significant difference between both incidences when subtracting the outbreak, i.e. most part of the time the number of detected cases was within the expected interval.

To ensure less transmission, the fulfilment of preventive measures such as hand washing and wearing masks is essential [27]. Using face masks has been associated with a lower incidence among indoor sports, and may be protective among outdoor sports with prolonged close contact between players [28]. Other implemented measures are regular symptoms monitoring and temperature measurement [17]. These strategies seem insufficient in young people, who mainly remain asymptomatic or with mild symptoms [1]. Therefore, regular testing is crucial. Usually among professional athletes, a negative test is needed to be able to play, but this condition is not required in the younger ones; so, our study was a good opportunity since it offered real-time results.

Previous studies, regarding the impact of the implementation of preventive protocols in sports, have demonstrated a reduction in the observed cases with respect to the expected [29]. Our study does not show fewer observed cases than expected, but the same. This suggests that despite its complexity in terms of transmission, protocols can be implemented in the sports centers to reduce the average in terms of infections. In this way, if they were improved, they would lead to safer environments.

Another important point observed is the different attack rate in the cohorts, since it was three times higher in the participants living in the Masia than in participants who lived at their own households (30% vs. 10%). The same difference was observed in the seven monitored teams (higher AR in those who came from RP1). In addition, AR was significantly higher in those who lived in the Masia compared to those who only worked there. This is related to the great outbreak derived from common life in the Masia, where transmission was easier due to the close contact during cohabitation and the difficulty in guaranteeing the safety distance and the use of face masks at all times. As is well known, a high level of indoor interactions can produce major outbreaks.

When analyzing the type of sport, lower transmission has been reported in outdoor and non-contact sports [28]. In our study, we saw a higher AR on a basketball team, an indoor and contact sport. However, no differences were found when analyzing the types of sport depending on the place of residence.

Going back to the outbreak, a possible explanation for it was that the initial case could have been infected outside the facilities (only one out of the five initially positive cases belonged to RP2); however, we did not have enough evidence to prove that this participant was the initial case. Other authors have found that the majority of cases in these settings are predominantly attributed to community contacts, rather than transmission during sports [30].

Finally, another consideration is the high level of detection inferred from seroconversion. The fact that only 22.5% of the seroconversions had not previously been detected is a particular low value. Underdetection seemed to be restricted to cases without symptoms or so mild that they were not reported. Antibody levels have been correlated with severity, with moderate and severe cases being the most likely to detect antibodies [31]. This points out to an almost complete detection of moderate cases and a large detection of mild cases with systematic weekly screening.

To conclude, it was relevant that nine patients maintained positive IgG along the study, without being vaccinated or re-infected. While some authors have described a serology duration of about 5 months [32], others have described a longer seropositive response in teenagers and in patients with severe symptoms [33].

### Limitations

We could not infer attack rates in all the teams, as we lacked information on the ones who did not want to participate and those who were lost in the follow-up. In addition, it is possible that the established interval between testing could have meant that not all asymptomatic positive cases were detected. On the other hand, the study population was heterogeneous (more men than women, different age groups, specific sports…), which could mean that the study could not be extrapolated to all sports settings. Finally, some participants were immunized against SARS-CoV-2, so serologies became no longer helpful to discern a past infection.

Given the variability of infectivity and severity associated with each of the SARS-CoV-2 variants, it is possible that if this study had been carried out in season 2021–2022, in which the Omicron variable predominates and the majority of population including children were vaccinated, the outcomes would have been different.

## Conclusions

Physical activities in stable teams, especially outdoors, are not associated with an increased risk of transmission of SARS-CoV-2, since there were the same observed cases than expected in the community. A high level of interactions in closed settings can increase the risk of transmission and lead to major outbreaks. To reduce this risk, compliance with preventive measures is essential.

## Supplementary Information

Below is the link to the electronic supplementary material.

Supplementary file1 (DOC 87 KB)

Supplementary file2 (DOC 51 KB)

Supplementary file3 (DOC 186 KB)

Supplementary file4 (DOC 98 KB)

## Data Availability

The datasets generated during and/or analyzed during the current study are available from the corresponding author on reasonable request.

## Abbreviations

AR: Attack rate
COVID-19: Coronavirus disease 2019
FCB: Fútbol Club Barcelona
NP Ag: Nasopharyngeal antigen
NP PCR: Nasopharyngeal polymerase chain reaction
RP: Recruitment pathway
SARS-CoV-2: Severe acute respiratory syndrome coronavirus 2
