# Supplementary material for: SARS-CoV-2 transmission in teenagers and young adults in Fútbol Club Barcelona’s Multidisciplinary Sports Training Academy
Source: Eur J Pediatr. 2023 Mar 14;182(5):2421–32. doi: 10.1007/s00431-023-04880-x (PMC10010953; doi:10.1007/s00431-023-04880-x)

**Supplementary file 1.**

Players who are participating in the Sant Joan de Déu Hospital study of COVID-19 in the facilities of the Fútbol Club Barcelona (FCB) have to attend the tests on the day that corresponds to each group.

Once the responsible doctor has the results of the tests, the COMMUNICATION OF A POSITIVE CASE must be performed as follows (see Figure 1 at the end):

- In the event that a positive case has been detected, the official communication will be send to the Medical Centre and the Masia Educational Team.
- The responsible doctor will in turn notify the person in charge of the technical area. The technical manager of each section must inform the families of:
  - The result of the test.
  - That Sant Joan de Déu Hospital will notify the Catalan Epidemiological Surveillance System.
  - That if the player has symptoms, he will be visited by a pediatrician at Sant Joan de Déu Hospital.
  - That if they have any medical questions, they can contact the doctor responsible for their section and team.
  - That if the player is a resident at The Masia: they can come to pick up their children (being close contact or positive). In no case is it an obligation, but it is a recommendation.
- On the other hand, the secretary must notify the technician responsible for the team of the positive or close contact player. It will be the coach/technical secretary who will contact the players to notify the result of the tests and inform that they have to confine themselves to their home/room.

When it comes to report the players who end the isolation, each case and sport will have to be studied case by case. In this case, the responsible doctor will be the one to inform the medical/sports department and residence (respectively) about the dates of ending the confination. It will be the technical part who informs the player.

In the case of La Masia residents, the criteria and the logistics established are as follows:

- Isolated positive cases or close contacts in quarantine will remain confined to their rooms. If the availability of rooms allows it, individually.
- They will eat in their room.
- They will be allowed to go out to the outdoor garden area at some time during the day, at a time set by the Masia Educational Team (this time will be when they do not coincide with any non-confined resident).
- They will go out in small groups, carrying FFP2 masks and keeping a safe distance from other people, not being able to go out at the same time the positive players with the isolated ones.
- In the event that there are two positive players from different sections and there are no free rooms to separate the roommates, the two positive players will be confined in the same room.
- Continuous and direct contact will be maintained with families and players to accompany them as much as possible in the isolation period.


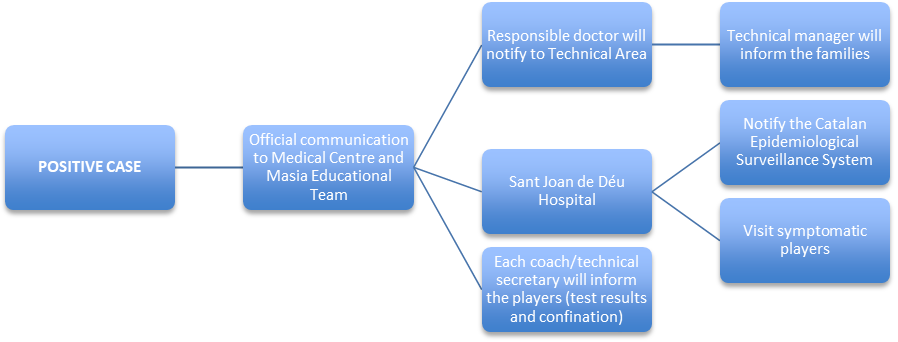

Supplement: Supplementary file 1 — Supplementary file1 (DOC 87 KB) [file 431_2023_4880_MOESM1_ESM.doc]
