# Supplementary material for: SARS-CoV-2 transmission in teenagers and young adults in Fútbol Club Barcelona’s Multidisciplinary Sports Training Academy
Source: Eur J Pediatr. 2023 Mar 14;182(5):2421–32. doi: 10.1007/s00431-023-04880-x (PMC10010953; doi:10.1007/s00431-023-04880-x)
Supplement: Supplementary file 2 — Supplementary file2 (DOC 51 KB) [file 431_2023_4880_MOESM2_ESM.doc]

**Supplementary File 2.**

The aim of this document is to establish the rules and preventive and health measures that will be carried out in the field of the Fútbol Club Barcelona (FCB) teams on their training, with the main objective of reducing the risk of transmission of SARS-CoV-2. This document may undergo modifications depending on the evolution of the epidemiological situation and the sanitary regulations.

The basic principles that will guide this document will be:

- Guarantee the safety and health of the players and the coaching staff.
- Guarantee the safety and health of all workers who interact with the players and the coaching staff.
- Guarantee the safety and health of the surroundings of the FC Barcelona facilities.
- Promote personal and collective protection and hygiene measures against the transmission of SARS-CoV-2.

This document will establish the guidelines for the return to training in the base teams, which will be carried out in 3 phases:

- Phase 1. Initial assessment
- Phase 2. Establishment of stable coexistence teams
- Phase 3. Training of stable coexistence teams

**Phase 1. Initial assessment**

Before the start of the date of the first training session, all the participants, players or coaching staff, must declare in a responsible manner that in the last 14 days:

- they have not had symptoms compatible with COVID-19
- they have not been diagnosed with the disease
- they have not lived with people who have been ill
- they have not had close contact with people affected by the disease.

Any participant who is suspected or confirmed to present the infection, or who has been close contact of a confirmed case in the last 14 days, will not be able to start the training and must communicate it to the responsible doctor and the COVID-19 cases medical manager as soon as possible.

**Phase 2. Establishment of stable coexistence teams**

To maintain safe environments and respecting current regulations, teams, defined as stable sport-specific groups (SSG), will be established. Teams are groups formed by athletes and coaching staff who, continuously, have close contact during the sports training, and cannot guarantee the interpersonal safety distance and the use of face mask.

In order to establish a team, none of its members should present symptoms of COVID-19 during the group's stabilization period, which is 14 days. Accordingly, special care measures should be taken during these first 14 days.

The establishment of the teams will prevent possible transmission of SARS-CoV-2 and will ensure the traceability of stable groups by identifying athletes and coaching staff, their symptoms and their possible risk contacts.

During the teams establishment period, the training activities should be carried out sequentially:

- 1st week: individual work respecting the interpersonal safety distance and avoiding physical contact.
- 2nd week: work phase in groups.

After the first 14 days, if there is no positive or suspicious case, or close contact, the team may be considered established and Phase 3 may start (see following section).

If, during this period, there is a positive case, close contact or someone showing symptoms, it should be reported immediately to the responsible doctor and the COVID-19 cases medical manager. Likewise, activities will be suspended and an analysis of the situation will be carried out to take the pertinent measures.

**Phase 3. Training of stable coexistence teams**

Once the teams have been established, the training activities can be carried out without maintaining of interpersonal safety distance and the strict use of face mask, assuming possible physical contact between the players. The coaching staff must maintain, as far as possible, the use of a face mask and interpersonal distance during training sessions.

Each team will have a defined training schedule and a determined changing room. Since the teams seek to limit the number of people affected in the case of having a positive case, it is highly recommended to not have interactions between different teams. In cases where a player has to carry out activities with a different team, the risk and need must be previously assessed by the doctor responsible for the section.

**General rules within the facilities**:

- An access point to the facilities will be established. At this point the player/staff will be identified, their data will be recorded and their body temperature will be taken using a laser thermometer.
  - If the temperature is ≤ 36.9ºC, the access will be allowed by giving an identification bracelet, which will be different every day, and which will indicate that that player/staff has successfully passed through the control point. Every player/staff must wear the bracelet at all times within the club's facilities. At the same access point, the player/staff must wear a hygienic mask and must clean his/her hands with hydroalcoholic solution.
  - In the event that the player/staff has a temperature ≥ 37ºC, entry to the facilities will be denied and he/she will be instructed to medical assessment by the responsible doctor from the Medical Centre. In cases when the Medical Centre is closed, the COVID-19 cases medical manager will be notified via telephone, who will make the pertinent indications and recommendations to carry out the medical evaluation.
- Before training, the players/staff must notify the section doctor or physiotherapist if they present any symptoms suggestive of COVID-19 infection (headache, cough, sneezing, mucus, sore throat, diarrhea, fever, loss of smell or loss of taste. In these cases, the person will not be able to start sports activities, will immediately leave the team and will be referred to the Medical Center for the corresponding evaluation. In the event that the Medical Center is closed, the case will be communicated to the COVID-19 cases medical manager, who will indicate the actions to be followed.
- The training will begin progressively team after team, which will allow avoiding a high concentration of people at the same place and time, and also will avoid crossing between the groups, as well as having a better traceability in case of having a suspicious positive case.
- The spaces for each team will be sectorized. Circulation circuits will be one-way and crossings with other teams will be avoided.
- Within the facilities, all persons must wear a mask, covering the mouth and nose, must maintain a minimum interpersonal distance of 1.5 meters and must use hydroalcoholic solutions.
- Within the facilities there will be posters and informative infographics of basic and essential hygiene measures.
- Family members or other people not included in the team will not be allowed in the training sessions.
- All the facilities where the trainings will take place and the material to be used will be well established. The facilities of each team will be for their exclusive use, and will be isolated from other people, with the exception of the essential personal for disinfection, cleaning, organization and surveillance.
- Daily specific cleaning and disinfection of the facilities will be carried out, both indoors and outdoors. The entire facility will be ventilated frequently.
- Only one player may be performing treatment in the physiotherapy room. Both the physiotherapist and the player must wear the mask at all times.
- No person may access, transit, use or remain in the medical area or the physiotherapy room except with the express permission of the responsible doctor, the COVID-19 cases medical manager, the responsible physiotherapist or the person in charge of the facility, in this order.
- Video rooms and common areas cannot be used.
- It won’t be allowed to eat or drink inside the facilities, except during training sessions. Each player must have his/her own bottle of water, which must be correctly identified.
- No items for personal use may be shared.

**Management of suspected and confirmed COVID-19 cases:**

- In the event that a player/staff presents symptoms compatible with COVID-19, the doctor in charge of the section and the COVID-19 cases medical manager must be notified immediately.
- No person with symptoms compatible with COVID-19 will be able to enter to the training facilities and will not be able to be in contact with their team, until they have the corresponding medical evaluation. The responsible physician, together with the COVID-19 cases medical manager, will decide where will be the assessment carried out.
- Suspected cases of COVID-19 infection must carry out the diagnostic test and must do home isolation pending the result of the test. At that time, the search for close contacts within the training environment, including the team, will begin.
  - If the test result is negative and clinical suspicion is low, infection will be ruled out and isolation may be finished.
  - In the event that the test result is positive, the isolation will be maintained, which will last up to three days after the resolution of the fever and the symptoms, with a minimum of 10 days from the onset of the symptoms. In asymptomatic cases, the isolation will be maintained until 10 days after the date of taking the sample for diagnosis.
- All the team that belongs to the positive case, as well as their close contacts of the 48 hours prior to the onset of symptoms or the confirmation of the test in the case of asymptomatic patients, must performed the corresponding diagnostic test and must do the corresponding home isolation until the result of the test.
  - If the test is negative, the isolation will be maintained for up to 14 days from the last contact.
  - If the test is positive, it will be considered a confirmed case and the corresponding actions must be carried out.
- If a player or staff is in close contact with a person with a confirmed infection who does not belong to the club environment, he/she must communicate this immediately to the responsible physician and the COVID-19 cases medical manager, and will not be able to enter to the training facilities or be in contact with his/her team, until the result of the diagnostic tests of the index case is known.
