# Supplementary material for: SARS-CoV-2 transmission in teenagers and young adults in Fútbol Club Barcelona’s Multidisciplinary Sports Training Academy
Source: Eur J Pediatr. 2023 Mar 14;182(5):2421–32. doi: 10.1007/s00431-023-04880-x (PMC10010953; doi:10.1007/s00431-023-04880-x)
Supplement: Supplementary file 3 — Supplementary file3 (DOC 186 KB) [file 431_2023_4880_MOESM3_ESM.doc]

**Supplementary File 3: Methods calculations**

With the aim of identifying when transmission was significant and led to local outbreaks, we determined the expected number of cases to be found in each screening according to the surrounding incidence. We used the number of diagnosed cases in Catalonia by age groups [<https://dadescovid.cat/descarregues?lang=eng>]. In our analysis, three different age groups were considered:
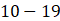
,
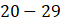
 and
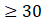
. The number of expected cases at time *t*,
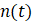
, was computed as:


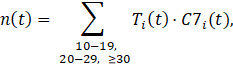


where
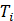
 is the number of screening tests for
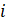
 age group at day t and
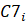
 is the number of cases detected among general population during the last seven days (t, t-1, …, t-6) for
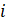
 age group divided by the population of Catalonia of this age group.

The probability to find
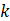
 positives,
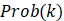
, was defined as:


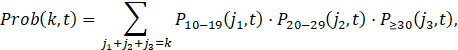


where
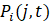
 is the probability to find
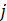
 positives in the
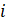
 age group, which can be computed as:


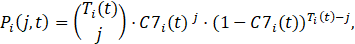


where
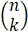
 is the binomial coefficient. This probability of finding a given number of positives in the general population was compared with the actual number of cases found in the FCB setting to assess and compare the levels of transmission.
