# Supplementary material for: SARS-CoV-2 transmission in teenagers and young adults in Fútbol Club Barcelona’s Multidisciplinary Sports Training Academy
Source: Eur J Pediatr. 2023 Mar 14;182(5):2421–32. doi: 10.1007/s00431-023-04880-x (PMC10010953; doi:10.1007/s00431-023-04880-x)
Supplement: Supplementary file 4 — Supplementary file4 (DOC 98 KB) [file 431_2023_4880_MOESM4_ESM.doc]

**Supplementary File 4: Pairwise comparisons of Attack Rates**

**Table 3.** Comparison of the attack rates (AR) between all the participants from recruitment pathway 1 (RP1) and recruitment pathway 2 (RP2).

*There were more cases of infection among participants from RP1 than among participants from RP2, the difference is significant (ODDs ratio 3.7 [1.8-7.6]).*

| **RP1 vs. RP2**  **(all the participants in the study)** | | | |  |  |  |  |
| --- | --- | --- | --- | --- | --- | --- | --- |
| **Cohort** | **n** | **Positives** | **Attack Rate** | **p-value** | **ODDs ratio** |  |  |
| RP1 | 70 | 21 | 30% | **<0.01** | **3.7059 [1.8101-7.5872]** |  | |
| RP2 | 164 | 17 | 10% | **<0.01** | **0.2698 [0.1318-0.5525]** |  | |
| *Total* | *234* | *38* | *16%* |  |  |  | |

**Table 4.** Comparison of the attack rates (AR) between workers and players (=residents) from RP1 (La Masia).

*There were more cases of infection among La Masia residents than among La Masia workers, the difference is significant (ODDs ratio 13.7 [1.8-107.1]).*

| **Workers and residents in La Masia (RP1)** | | | |  |  |
| --- | --- | --- | --- | --- | --- |
| **Cohort** | **n** | **Positives** | **Attack Rate** | **p-value** | **ODDs ratio** |
| Worker | 33 | 1 | 3% | **<0.01** | **0.0729 [0.0093-0.5692]** |
| Resident | 70 | 21 | 30% | **<0.01** | **13.7143 [1.7568-107.0601]** |
| *Total* | *103* | *22* | *21%* |  |  |

**Table 5.** Comparison of the attack rates (AR) between the participants from the seven complete monitored teams that belonged to RP1 vs. RP2.

*Among participants from the seven complete monitored teams, the AR was significantly higher in those who live in La Masia (RP1) compared to those who do not reside there (RP2), with a p-value < 0.01, and ODDS 3.6 [1.6-8.2].*

| **RP1 vs. RP2**  **(only participants from the 7 complete monitored teams)** | | | |  |  |
| --- | --- | --- | --- | --- | --- |
| **Cohort** | **n** | **Positives** | **Attack Rate** | **p-value** | **ODDs ratio** |
| RP1 (La Masia) | 43 | 15 | 35% | **<0.01** | **3.5714 [1.5583-8.1850]** |
| RP2 | 115 | 15 | 13% | **<0.01** | **0.2800 [0.1222-0.6417]** |
| *Total* | *158* | *30* | *19%* |  |  |

**Table 6.** Comparison of the attack rates (AR) between all the participants from the seven complete monitored teams (from both RP1 and RP2).

*The Senior youth basketball team had the highest AR among all complete monitored teams, with a p-value <0.01 and an ODDS ratio of 12.3 [3.8-39.7]. Among the rest of the monitored teams there were no significant differences in terms of AR.*

| **7 complete monitored teams** | | | | |  |  |
| --- | --- | --- | --- | --- | --- | --- |
| **Cohort** | | **n** | **Positives** | **Attack Rate** | **p-value** | **ODDs ratio** |
| Men Outdoor Football | Senior youth A | 35 | 5 | 14% | 0.4763 | 0.6533 [0.2301-1.8552] |
|  | Senior youth B | 33 | 8 | 24% | 0.4543 | 1.4982 [0.5973-3.7580] |
|  | Junior youth A | 26 | 2 | 8% | 0.1691 | 0.3095 [0.0690-1.3895] |
| Women Outdoor Football | Reserve | 21 | 2 | 10% | 0.3705 | 0.4098 [0.0901-1.8643] |
|  | Senior youth | 13 | 1 | 8% | 0.4647 | 0.3333 [0.0416-2.6686] |
| Basketball | Reserve | 15 | 2 | 13% | 0.7380 | 0.6319 [0.1348-2.9620] |
|  | Senior youth | 15 | 10 | 67% | **<0.01** | **12.3000 [3.8064-39.7464]** |
| *Total* | | *158* | *30* | *19%* |  |  |

**Table 7.** Comparison of the attack rates (AR) between the participants from the seven complete monitored teams that belonged to RP1 (La Masia).

*There were no significant differences in ARs between the RP1 participants from the seven complete monitored teams (no differences between residents in La Masia depending on which team/sport they played).*

| **Participants from the 7 complete monitored teams, that live at La Masia (RP1)** | | | | |  | |  | |  |
| --- | --- | --- | --- | --- | --- | --- | --- | --- | --- |
| **Cohort** | | **n** | **Positives** | **Attack Rate** | | **p-value** | | **ODDs ratio** | |
| Men Outdoor Football | Senior youth A | 8 | 2 | 25% | | 0.6916 | | 0.5641 [0.0989-3.2171] | |
|  | Senior youth B | 20 | 7 | 35% | | 1.0000 | | 1.0096 [0.2872-3.5494] | |
|  | Junior youth A | 5 | 0 | 0% | | 0.1449 | | NA | |
| Women Outdoor Football | Reserve | 0 | 0 | NA | | NA | | NA | |
|  | Senior youth | 0 | 0 | NA | | NA | | NA | |
| Basketball | Reserve | 2 | 1 | 50% | | 1.0000 | | 1.9286 [0.1120-33.2069] | |
|  | Senior youth | 8 | 5 | 63% | | 0.1036 | | 4.1667 [0.8343-20.8093] | |
| *Total* | | *43* | *15* | *35%* | |  | |  | |

**Table 8.** Comparison of the attack rates (AR) between the participants from the seven complete monitored teams that belonged to RP2.

*Among the RP2 participants (who did NOT live in La Masia), from the seven complete monitored teams, the team with a higher AR with p-value < 0.01 was the Senior youth basketball team, with an ODDS of 24.5 [4.2-143.0]. Among the rest of the RP2 participants, there were no significant AR differences.*

| **Participants from the 7 complete monitored teams, that live at their own homes (RP2)** | | | | |  | |  |  |
| --- | --- | --- | --- | --- | --- | --- | --- | --- |
| **Cohort** | | **n** | **Positives** | **Attack Rate** | **p-value** | **ODDs ratio** | | |
| Men Outdoor Football | Senior youth A | 27 | 3 | 11% | 1.0000 | 0.7917 [0.2061-3.0410] | | |
|  | Senior youth B | 13 | 1 | 8% | 1.0000 | 0.5238 [0.0631-4.3487] | | |
|  | Junior youth A | 21 | 2 | 10% | 0.7350 | 0.6559 [0.1364-3.1535] | | |
| Women Outdoor Football | Reserve | 21 | 2 | 10% | 0.7350 | 0.6559 [0.1364-3.1535] | | |
|  | Senior youth | 13 | 1 | 8% | 1.0000 | 0.5238 [0.0631-4.3487] | | |
| Basketball | Reserve | 13 | 1 | 8% | 1.0000 | 0.5238 [0.0631-4.3487] | | |
|  | Senior youth | 7 | 5 | 71% | **<0.01** | **24.5000 [4.1975-143.0026]** | | |
| *Total* | | *115* | *15* | *13%* |  |  | | |
